# Uneven Protection at Work During COVID-19: Union Voice, Paid Sick Leave and Safety Regimes in Ontario

**DOI:** 10.1177/0160449X261463608

**Published:** 2026-07-10

**Authors:** Mohammad Ferdosi

**Affiliations:** 1 École de Relations Industrielles, 7984 Université de Montréal, Montréal, Canada

**Keywords:** Ontario, unions, worker voice, occupational health and safety, COVID-19

## Abstract

Using the employed subsample of a broader Ontario-wide online survey, this article compares union and non-union experiences of COVID-19 safety in 2020. We examine infection control, paid time off, anxiety, and awareness of union advocacy, using this non-probability sample for analytic generalization rather than population estimates. In this sample, union-covered respondents more often reported visible ventilation and filtration, referenced joint committees or designated representatives in their accounts, and reported more usable paid sick leave, while stress and anxiety were high in both groups, and differences by union coverage were small within this sample. We discuss what these patterns suggest for unions, employers, and policy as temporary COVID-19 leave has ended and long-term protections are debated.

## Introduction

The COVID-19 pandemic made the workplace a central site of both risk and protection for workers in Ontario. Many higher-wage office workers shifted to remote or hybrid arrangements while workers in health care, long-term care, education, retail, warehousing, transport, and cleaning continued to spend their days in shared indoor spaces. Early evidence showed that outbreaks clustered in sectors with close contact, crowded rooms, and limited ability to separate staff and that workplace clusters seeded infections in surrounding communities (Murti et al. 2021; Buchan et al. 2022; Sritharan et al. 2025). Even into 2023, working from home remained concentrated in higher-wage computer-based occupations (Messacar, Morissette and Deng 2020; Mehdi and Morissette 2021; Statistics Canada 2024*b*), so those who stayed home versus on-site tended to reflect existing inequalities in income, security, and control over working conditions.

Ontario is a useful setting for examining how worker voice matters for safety during a pandemic. The province relies on an internal responsibility system that expects employers and workers to identify hazards and control risks through joint health and safety committees (Walters and Nichols 2007; Hall and Tucker 2022). When these committees are active and resourced, they can improve compliance and reduce uncertainty (Lewchuk, Robb and Walters 1996; Nichol et al. 2020). When committees exist mostly on paper, they leave large gaps between formal policies and day-to-day practice, and workers may be uncertain about who speaks for them or what happens when they raise concerns (Barnetson 2010). Unions play a key role in this system since they nominate worker representatives, train stewards, and provide channels for escalation when committee processes stall or employers resist change (Freeman and Medoff 1984; Walters and Nichols 2007).

Ontario's internal responsibility system is operationalized through legal requirements for worker participation. Many workplaces must establish a joint health and safety committee (JHSC) with worker and management representatives, while smaller workplaces must have a health and safety representative (Government of Ontario 2017). JHSCs are typically required in workplaces with 20+ regularly employed workers, with at least half of the members representing workers (Ontario 1990; Government of Ontario 2017). Committees conduct inspections, make recommendations, and provide a channel for documenting and escalating hazards, with employers required to respond in writing to written recommendations within 21 days (Ontario 1990). Ontario's system is also underpinned by statutory worker rights to participate in health and safety processes and to refuse unsafe work, with Ministry of Labor inspectors serving as an external enforcement backstop when internal mechanisms fail. In unionized workplaces, worker-side JHSC members are commonly union-nominated and supported through steward networks and training. These features matter for pandemic safety because they shape whether concerns about masking, staffing, and ventilation are handled through an institutional process rather than isolated individual complaints.

The pandemic also exposed the centrality of paid sick leave to any credible strategy for infection control. Access to paid sick leave in Canada is uneven and strongly structured by union coverage, sector, and wage level (MacIsaac and Morissette 2023; Statistics Canada 2024*a*; Statistics Canada 2024*c*). During the first year of COVID-19, Ontario workers navigated a patchwork of employer policies, federal income supports, and collective agreement provisions (Government of Ontario 2024; Government of Canada 2022; Employment and Social Development Canada 2022; Tucker and Vosko 2021). Workers with paid time off could usually isolate or stay home when symptomatic without immediate financial penalty. Workers without such entitlements faced what many described as an income test rather than a safety rule (Quinlan, Mayhew and Bohle 2001; Vosko 2006; Noack and Vosko 2011).

Scholars of labor, health, and employment have documented how COVID-19 magnified existing inequalities in exposure, job security, and mental health. Research on health care, long-term care, education, and food processing links the perceived adequacy of personal protective equipment, ventilation, and staffing to anxiety and burnout (Smith et al. 2020; Smith et al. 2022; Buchan et al. 2022). Studies of income support programs and sick leave connect more generous and accessible benefits with lower rates of presenteeism and reduced transmission (Pichler, Wen and Ziebarth 2020; MacIsaac and Morissette 2023). Work on precarious employment and fissured workplaces highlights that even strong formal rights can be hard to exercise when jobs are unstable, hours unpredictable, and the threat of replacement is ever-present (Quinlan, Mayhew and Bohle 2001; Lewchuk 2017; Weil 2014; Vosko et al. 2020). However, existing research tends to examine these dimensions separately (union representation, employment precarity, sick leave, or ventilation standards) rather than as interacting configurations that workers experience as more or less protective “safety regimes.”

This article builds on that scholarship but asks a different question. We ask how different institutional configurations of union representation, workers’ accounts of joint health and safety processes (e.g., committees/representatives), and paid sick leave were reflected in what protection looked and felt like on the ground. We focus on how these institutions combined, in respondents’ accounts, into more or less protective safety regimes for Ontario workers who remained employed during the first pandemic year. We draw on an Ontario-wide online survey carried out in late 2020, combining closed-ended questions with rich open-ended accounts to examine how workers in different institutional settings interpreted and navigated COVID-19 risks at work and how formal rules translated into everyday practice (Smith et al. 2015).

We ask three questions: (1) how reported infection control measures such as masking rules, ventilation or filtration actions and distancing practices varied by union coverage; (2) how access to paid sick leave and other paid time off figured in workers’ accounts of decisions about going to work while symptomatic, exposed or anxious; and (3) how workers’ accounts of collective voice (including unions and joint health and safety processes) intersected with material supports and employment precarity in respondents’ accounts of perceived safety, stress and conflict in the workplace.

The analysis aims to contribute to labor studies in three ways. It brings together the literature on union voice, precarious employment, and occupational health to develop a framework for understanding what we call safety regimes at work. It offers a cross-sector comparison of union and non-union workers’ experiences during the first pandemic year in Ontario, with attention to how workers themselves narrate union advocacy and committee work. It also treats the convenience sample as an opportunity for analytic generalization, using the survey to identify recurring configurations of protection and vulnerability and to link them to concrete practices that unions, employers, and regulators can adopt.

## Literature Review

Work on unions and worker voice has long argued that collective representation can convert individual grievances into enforceable rules and ongoing problem-solving. In classic formulations, unions provide an institutional channel for voice that can reduce quit rates and raise productivity when they institutionalize feedback from the shop floor (Hirschman 1970; Freeman and Medoff 1984). Subsequent research emphasizes that voice is not a single mechanism. It includes formal bargaining, grievance procedures, joint committees, and less formal practices of representation and support through which stewards and activists interpret rules and advise members (Walters and Nichols 2007; Lewchuk 2017).

For occupational health and safety scholars, joint health and safety committees are a key site where this collective voice is expected to operate. These committees can investigate hazards, recommend controls, and monitor follow-up. Studies of joint committees in Ontario and other jurisdictions find that they are most effective when worker members are supported by unions, have training time, and have clear routes to escalate unresolved issues, including the ability to trigger inspections or enforcement (Lewchuk, Robb and Walters 1996; Walters and Nichols 2007; Nichol et al. 2020; Hall and Tucker 2022). At the same time, research on the internal responsibility system in Ontario shows that joint committees sometimes exist only on paper, that workers may not know who their representatives are, and that employer dominance or the threat of reprisal can blunt committee action (Walters and Nichols 2007; Hall and Tucker 2022; Barnetson 2010). Comparative studies emphasize that committees are most effective when backed by strong legal rights, credible enforcement, and broader capacity to mobilize members. Without these supports, committees can become forums for information sharing without real power to demand changes in how work is organized (Walters and Nichols 2007; Barnetson 2010). In Ontario, these statutory participation mechanisms are anchored in the Occupational Health and Safety Act and the internal responsibility system, making committees/representatives a central institutional pathway through which worker voice is expected to operate (Ontario 1990; Government of Ontario 2017).

A second body of scholarship focuses on precarious employment, fissured workplaces, and the ways that labor market insecurity undermines enforcement of employment standards. Precarious work is characterized by insecure contracts, variable hours, low wages, and limited access to benefits or career ladders (Vosko 2006; Noack and Vosko 2011; Lewchuk 2017). In such settings, hazards are often more frequent and severe. Workers are more likely to be exposed to physically demanding or repetitive tasks, to work irregular schedules, and to lack the bargaining power needed to refuse unsafe work (Quinlan, Mayhew and Bohle 2001; Smith et al. 2015). Studies in Canada and internationally link precarious employment to higher rates of injury, poorer health, and weaker access to safety training and equipment (Quinlan, Mayhew and Bohle 2001; Barnetson 2010; Lewchuk 2017). These patterns reflect both employer practices and the broader regulatory environment that allows risks to be shifted onto workers with fewer resources, especially in fissured workplaces where subcontracting and agency work fragment responsibility (Weil 2014; Vosko et al. 2020; Vosko, Tucker and Casey 2019). Recent Ontario evidence using workers’ compensation claims and labor force data links precarious employment to higher risks of lost time occupational injury or illness and to work-related COVID-19 infections in compensation claims (Shahidi et al. 2024*a*; Shahidi et al. 2024*b*), supporting our focus on precarity as a key constraint on usable protection at work.

Research on the COVID-19 pandemic has built on these insights. Analyses of outbreaks in Ontario and other provinces show that infections were concentrated in in-person, indoor, close-contact sectors such as long-term care, health care, and food processing (Murti et al. 2021; Buchan et al. 2022; Sritharan et al. 2025). Many of these jobs are lower wage, have high proportions of racialized and migrant workers, and are structured through temporary contracts or subcontracting chains that weaken workers’ bargaining power (Noack and Vosko 2011; Vosko, Tucker and Casey 2019). Studies of health care and education workers in Canada report that perceived adequacy of personal protective equipment, staffing, and infection control procedures is closely linked to symptoms of anxiety, depression, and burnout (Smith et al. 2020; Smith et al. 2022). These findings suggest that mental health outcomes during the pandemic are closely tied to material conditions in the workplace.

A related strand of work examines paid sick leave and income support programs as public health tools. Evidence from Canada, the United States, and Europe shows that workers with access to paid sick days are less likely to attend work while ill, more likely to comply with isolation guidance, and more able to manage household responsibilities during school or childcare closures (Pichler, Wen and Ziebarth 2020; MacIsaac and Morissette 2023; Tucker and Vosko 2021). Studies of emergency sick leave programs introduced during COVID-19 suggest that generous and easily accessible benefits reduced transmission and flattened epidemic curves (Pichler, Wen and Ziebarth 2020; Government of Canada 2022). Analyses of provincial and federal income supports in Canada show that benefit design created a two-tier system where workers who qualified for federal benefits received incomes closer to replacement levels while workers tied to provincial social assistance programs remained in deep poverty and often lacked the means to comply with public health advice (Tucker and Vosko 2021; Government of Ontario 2024).

The emerging literature on unions and COVID-19 finds that unions played multiple roles. They bargained over hazard pay, staffing, and redeployment, advocated for stronger public health measures and paid leave at the legislative level, and helped members navigate complex and shifting policies around isolation, testing, and vaccination (Hall and Tucker 2022; Hopwood et al. 2022; Tucker and Vosko 2021). Case studies in sectors such as health care, education, and transportation suggest that union presence often coincided with clearer infection control routines and more predictable access to leave, although unions operated within tight constraints when employers and governments relied on emergency powers or when staffing shortages undermined enforcement (Hall and Tucker 2022; Vosko et al. 2020; Smith et al. 2020).

Several contributions point to the importance of indoor air quality and ventilation during the pandemic. As evidence accumulated on airborne transmission, building scientists and public health agencies converged on a layered control strategy that included improved ventilation, higher grade filtration, use of portable air cleaners where central systems were weak, masking or respirator use where needed and policies that minimized the time people spent together in poorly ventilated rooms (Morawska and Milton 2020; Bazant and Bush 2021; Public Health Ontario 2022; Public Health Agency of Canada 2024; Marr and Samet 2024). These recommendations are reflected in evolving standards for schools, health care facilities, and other public buildings, yet many workers reported that ventilation remained opaque and that they could not tell whether systems met recommended targets or who was responsible for maintaining equipment (Hall and Tucker 2022).

This article develops a framework for what we call safety regimes during COVID-19 to move beyond one-variable explanations (e.g., “union vs non-union” or “ventilation vs no ventilation”). For a respiratory pandemic, workplace protection is best understood as a configuration of institutional resources and governance capacities that shapes whether risk controls are implemented as routine practice, whether workers can comply with public health guidance without absorbing the full cost individually, and whether concerns can be raised and resolved without individualized retaliation. Each dimension addresses a different coordination problem—reducing exposure (material controls), enabling compliance without income loss (paid leave), and translating guidance into enforceable routines (collective voice)—and none is reliably protective on its own when the others are weak.

We specify three interacting dimensions. First, material controls that reduce exposure at source and in shared air (e.g., remote/hybrid arrangements where feasible, ventilation/filtration, masking routines where needed, workflow and staffing adjustments). Second, temporal security through paid sick leave/paid time off that makes isolation, testing, or recovery feasible without immediate income loss, reducing the pressure toward presenteeism (Pichler, Wen and Ziebarth 2020; Tucker and Vosko 2021; MacIsaac and Morissette 2023). Third, collective voice through unions and statutory participation mechanisms (e.g., joint committees/representatives) that translates evolving guidance into workplace routines and compliance expectations and provides credible escalation when implementation is contested or uneven (Walters and Nichols 2007; Hall and Tucker 2022). Precarious and fissured employment is treated as a cross-cutting constraint that can erode each dimension in practice, by limiting access to leave, weakening the ability to refuse unsafe work, or excluding temporary/agency workers from committee channels, even within otherwise well-resourced workplaces (Quinlan, Mayhew and Bohle 2001; Weil 2014; Vosko, Tucker and Casey 2019).

This specification is also aligned with what the study can observe with integrity. The survey directly captures multiple infection-control measures and access to paid leave, while open-ended accounts repeatedly describe whether workers experienced credible voice and escalation channels through unions, joint committees/representatives, or designated advocates. Other pathways, such as safety culture, psychosocial climate, or managerial professionalism, are plausibly important, but in this dataset, they cannot be separated as distinct explanatory dimensions for systematic comparison; to the extent they matter here, respondents largely encounter them through whether controls are implemented and maintained, whether leave is usable, and whether voice is credible. The framework, therefore, foregrounds institutional levers that are visible in workers’ accounts and actionable in labor and policy debates about worker protection in crisis periods.

## Methods

The data come from a broader anonymous online survey conducted between August and December 2020 by a university-based research team in partnership with a community anti-poverty organization. The parent survey was open to Ontario residents aged 18 years or older who were not retired. Although eligibility was broad, the study was designed to assess how COVID-19 affected low-income and working individuals throughout Ontario. Consistent with that purpose, the questionnaire included modules on government programs and services, employment before and during COVID-19, workplace dynamics and safety, health, housing, food security, financial well-being, community resources, and views of government.

Because there was no feasible sampling frame for a province-wide survey of this kind and resources for a large commercial survey were limited, the research team used a convenience and snowball recruitment strategy. Eligible participants were recruited between August and December 2020 through social media and labor and community networks associated with the broader project. These recruitment channels were consistent with the study's aim of reaching low-income and working Ontarians, but they also mean that the sample should not be understood as representative of Ontario residents or Ontario workers. This strategy yielded 833 respondents who completed at least one section of the questionnaire.

For the analysis in this article, we focus on the subset who reported working for pay at some point during 2020 and who indicated whether they were covered by a union. In other words, the broader survey was not restricted to workers, but this article analyzes the employed subsample because its research questions concern workplace protection, paid leave, and worker voice. This group includes 440 respondents, of whom 162 reported union coverage and 278 did not. Respondents worked across the public sector, the private for-profit sector, and the private not-for-profit sector; the survey also captured a more granular “type of work” category (e.g., K-12 education, health care, finance/insurance, retail/wholesale, construction, manufacturing), which we use descriptively to assess whether more protective configurations clustered in particular work settings. Descriptive characteristics of the analytic sample overall and by union coverage are presented in Table 1. Unless otherwise noted, all quantitative analyses in this article use this same 440-respondent analytic sample; denominators vary across measures because of item non-response.

**Table 1.** Characteristics of the Analytic Sample Overall and by Union Coverage.

Characteristic	Total (*n* = 440)	Union-Covered (*n* = 162)	Non-Union (*n* = 278)
Age group	*n* = 439	*n* = 162	*n* = 277
18–34	92 (21.0)	30 (18.5)	62 (22.4)
35–54	220 (50.1)	80 (49.4)	140 (50.5)
55+	127 (28.9)	52 (32.1)	75 (27.1)
Gender	*n* = 439	*n* = 161	*n* = 278
Female	297 (67.7)	108 (67.1)	189 (68.0)
Male	135 (30.8)	51 (31.7)	84 (30.2)
Other	7 (1.6)	2 (1.2)	5 (1.8)
Race/ethnicity	*n* = 433	*n* = 158	*n* = 275
White	365 (84.3)	135 (85.4)	230 (83.6)
Racialized/other	60 (13.9)	22 (13.9)	38 (13.8)
Indigenous	8 (1.8)	1 (0.6)	7 (2.5)
Sector worked in before COVID-19	*n* = 402	*n* = 156	*n* = 246
Public sector	195 (48.5)	119 (76.3)	76 (30.9)
Private for-profit sector	140 (34.8)	18 (11.5)	122 (49.6)
Private not-for-profit sector	67 (16.7)	19 (12.2)	48 (19.5)
Employment arrangement during COVID-19	*n* = 435	*n* = 160	*n* = 275
Permanent full-time (30+ hrs/week)	253 (58.2)	117 (73.1)	136 (49.5)
Permanent part-time/variable full-time	68 (15.6)	18 (11.2)	50 (18.2)
Temporary/fixed-term/casual	66 (15.2)	21 (13.1)	45 (16.4)
Self-employed	48 (11.0)	4 (2.5)	44 (16.0)
Essential worker during COVID-19	*n* = 405	*n* = 148	*n* = 257
Yes	170 (42.0)	70 (47.3)	100 (38.9)
No	235 (58.0)	78 (52.7)	157 (61.1)

Note: Entries are *n* (%). Percentages are calculated using non-missing responses as the denominator within each column, so denominators vary across characteristics because of item non-response. Union coverage is based on whether respondents reported being covered by a collective agreement at their main job during COVID-19. Because sector was asked in the pre-COVID employment module, sector refers to respondents’ reported sector before COVID-19. “Racialized/other” combines non-white racialized categories and “Other” because of small cell sizes.

The sample is not representative of Ontario workers. Public sector workers and union members are overrepresented and racialized workers are underrepresented. Recruitment through unions, labor councils, and community organizations may have overrepresented respondents who are more engaged with workplace advocacy, including those in representative roles (e.g., stewards, health and safety representatives, JHSC members) or otherwise oriented toward raising concerns. Because the survey did not measure activism, union roles, or committee participation, we cannot assess this directly; accordingly, we interpret patterns as descriptive and use the sample for analytic rather than population generalization. We therefore treat the survey as an exploratory window into the experiences of a diverse group of workers and lean on analytic rather than statistical generalization (Smith et al. 2015).

The survey included several measures that are central to this article. Union coverage was measured with a question that asked whether the respondent was covered by a collective agreement at their main job. To capture workplace health and safety measures, respondents were asked whether their employer had implemented actions such as working from home arrangements, provision of personal protective equipment, installation of physical barriers, changes to ventilation or filtration, enhanced cleaning and screening of workers or customers. The survey did not include a closed-ended item measuring the presence or activity of a joint health and safety committee; references to committees/representatives come from respondents’ open-ended comments. Respondents were also asked how safe they felt going into work during COVID-19 compared to before and whether their stress or anxiety at work had increased, stayed the same, or decreased. Access to paid time off was measured through questions about paid sick days and other paid leave, including whether workers felt they could use these days when they had symptoms or an exposure without fear of reprisal. The questionnaire also recorded basic characteristics such as sector, occupation, and employment status to place responses in context.

To describe how union coverage, safety measures, and paid leave co-occurred in this non-probability sample, we constructed simple cross-tabulations of key indicators by union status and, where numbers allowed, by broad sector groupings. Given the non-probabilistic nature of the sample and the recruitment strategy, we did not apply weights, did not report confidence intervals, and did not fit statistical models. Instead, we focus on descriptive patterns that are substantively large and internally consistent within the sample, and we note where they are descriptively consistent with external administrative and survey evidence on paid sick leave coverage, working from home and sectoral risks in Ontario (Buchan et al. 2022; MacIsaac and Morissette 2023; Statistics Canada 2024*a*; Statistics Canada 2024*b*). The goal is to identify plausible mechanisms and configurations of protection that can inform theory and guide future research using more representative data rather than to estimate provincial prevalence.

The survey generated a substantial qualitative corpus. Nearly 360 respondents wrote open-ended comments about their work and daily lives during the pandemic. For this article, we focus on comments related to workplace safety, paid time off, union advocacy, and mental health. The research team read all comments and used an iterative process to identify recurring themes. Initial coding captured topics such as infection control routines, ventilation and air quality, staffing and workload, experiences with paid or unpaid leave, conflict with customers over masking or distancing, and interactions with unions, supervisors, and joint committees. Coding was then refined into a smaller set of themes that map onto the safety regime framework, with attention to how material protections, temporal security, and voice combined in different workplaces. We draw on the full set of comments to identify patterns that recur across respondents rather than relying on a small number of exceptional cases. We treat the comments as interpretive accounts that help us understand how workers made sense of the formal measures captured in the survey items and how they navigated tensions between safety, income needs, and workplace authority.

The mixed methods design allows us to connect patterns in the survey data with the narratives workers provide. Quantitative indicators of safety measures and leave entitlements can be read alongside comments that describe how these protections worked in practice and where they failed. This triangulation of descriptive patterns, qualitative accounts, and external evidence helps situate the configurations we describe as recurring themes in this sample rather than isolated anecdotes, and it provides a basis for the safety-regime typology developed in the findings and discussion.

## Findings

The findings are organized around the three dimensions of the safety regime framework. First, we describe overall patterns of perceived safety and stress at work during COVID-19. We then describe within-sample differences in reported infection control routines, including ventilation/filtration actions, by union coverage, and summarize how open-ended accounts depict the presence (or absence) of joint committees/worker representatives in daily COVID-19 safety practices. Finally, we consider how access to paid sick leave and other paid time off appeared in respondents’ accounts of decisions to attend work while ill or exposed, and how these material supports intersected with worker voice and precarity. Because the sample is non-probabilistic and we do not test statistical significance, the patterns below are presented as within-sample descriptive contrasts and should not be read as population estimates or causal effects. As shown in Table 1, union-covered respondents in this sample were concentrated more heavily in the public sector and in permanent full-time employment, while non-union respondents were more concentrated in the private for-profit sector and were more likely to report self-employment or other non-standard employment arrangements.

### Perceived Safety and Stress

Across this analytic sample, workers described the first pandemic year as a period of heightened risk and strain. More than two-thirds reported feeling somewhat or much less safe going into work during COVID-19 than before, and nearly four-fifths reported increased stress or anxiety at work. Almost half said they were pessimistic about their job or career prospects because of the pandemic. Within this sample, these signals were similar across union-covered and non-union respondents and echo sectoral studies that document heavy psychological burdens among health care workers, educators, and other frontline staff (Smith et al. 2020; Smith et al. 2022; Buchan et al. 2022).

Comments show how quickly infection risk became part of the daily texture of work. One education worker said that she had “started counting how many times I sanitized a door handle” and that it felt “like my whole job became trying not to get sick while still doing my real job.” A hospital worker described entering the building as “walking into a fog I cannot see and hoping I do not carry it home.” These accounts show how risk management became a second, unpaid layer of labor that sat on top of core tasks.

An early feature of the pandemic was the rapid reorganization of where work was done. Many higher-wage professional and administrative workers in our sample reported shifting to working from home for at least part of their time, while workers in health care, long-term care, retail, food service, transportation, cleaning, and manufacturing remained on site. Respondents who worked remotely most of the time generally reported lower exposure anxiety and more control over their physical environment but also feelings of isolation, blurred boundaries, and heavier workloads. One hybrid office worker wrote that she felt “lucky to be at home but also like I now live at work” because the workday had become “an endless stream of emails and video calls.”

On-site workers often felt that policies were written with office jobs in mind and did not translate well to crowded break rooms, vehicles, or shop floors. A non-union retail worker described being treated as essential but “not treated as worth protecting” and noted that she was denied the option to use banked sick time to stay home during the first wave. She summed up the experience as being “essential enough to get exposed but not essential enough to stay home with pay.” These experiences also illustrate the first element of safety regimes, namely, whether workers can control where they are physically located during a respiratory pandemic.

Within this 440-respondent analytic sample, union coverage was far more common in the public sector (61%; 119/195) than in the private for-profit sector (13%; 18/140), with intermediate coverage in the private not-for-profit sector (28%; 19/67). Sector was reported by 402 respondents in this subsample, and other items (e.g., working from home and specific employer controls) also have item non-response; accordingly, denominators vary across the descriptive comparisons below.

These sector and occupation differences matter for interpreting within-sample union/non-union contrasts because union coverage and the feasibility of remote work are unevenly distributed across work settings. Public sector respondents were more likely to report that their employer allowed working from home (78%; 141/181) than private sector respondents (57%; 64/113), reflecting the concentration of remote-eligible work in some settings and sustained in-person work in others. Using the survey's more granular “type of work” categories, open-ended accounts from respondents in sustained in-person settings (e.g., K-12 education and government health care) more often emphasized risk reduction in terms of layered on-site controls (e.g., personal protective equipment, enhanced cleaning, training, distancing routines, signage), whereas respondents in more office-based settings (e.g., finance/insurance and some postsecondary roles) more often described reduced exposure primarily through working from home. By contrast, retail/wholesale and food/hospitality respondents more often described continued in-person work alongside less consistent implementation of multiple on-site controls. We return to these work-setting differences when describing infection-control routines, paid leave, and worker voice, and we avoid reading these patterns as population estimates, given the convenience sample and item non-response. Within this sample, the more protective configurations were described most often in public sector and other large organization settings with established safety infrastructure, while thinner regimes were described more often in retail/food and some warehousing/logistics settings; however, these patterns are descriptive and confounded by the uneven distribution of union coverage and remote-work feasibility across sectors.

### Union Coverage and Infection Control Routines

In this sample, union-covered respondents more often described the visibility and consistency of infection control routines. Both union and non-union respondents reported that employers introduced measures such as hand sanitizer, cleaning protocols, some form of distancing, and masking policies. In this sample, union-covered workers more often described clear procedures and straightforward ways to raise concerns when protocols were not followed. Many pointed to joint health and safety committees or worker representatives who conducted walk-through inspections, tracked issues, and followed up on outstanding items. Stewards in some workplaces printed and posted simple checklists near entrances or in break rooms and kept copies of the most recent protocol so that workers could show them to supervisors if instructions were inconsistent.

A unionized cleaner in a hospital explained that once the joint committee simplified the protocols “into a one page checklist on every door, the fights on each shift dropped.” In her words, “before that it was you did not clean this or I already did that, after the checklist we could point to the paper and say here is what has to be done and who did it.” This is a concrete example of voice converting general guidance into an enforceable routine, in line with research that shows committees are most effective when their work is visible and tied to specific tasks (Lewchuk, Robb and Walters 1996; Walters and Nichols 2007; Nichol et al. 2020; Hall and Tucker 2022).

In this sample, non-union respondents were less likely to report this kind of stable routine and more likely to describe what we term thin procedural regimes. Many reported rules that shifted with management turnover or staffing changes. A warehouse employee without union coverage wrote that masks were “strictly enforced on the day shift and basically optional at night” because different supervisors had different views. She recounted being told one week that windows had to stay closed for security reasons and another week that they should be opened for air, and concluded that “there was no one to appeal to, you just did what each manager wanted.” Without a committee or representative to appeal to, workers relied on personal strategies such as rearranging tasks, avoiding crowded zones, or quietly wearing higher-grade masks. A retail worker put it this way and said that “the rule was whatever your manager felt like that day.”

These contrasts illustrate the second dimension of safety regimes. In respondents’ accounts, more protective regimes showed committees and stewards turning public health guidance into simple routines that reduce conflict and uncertainty. In less protective regimes, infection control depends on individual supervisors and informal workarounds.

### Ventilation, Transparency, and Trust

Ventilation and filtration became central topics as knowledge about airborne transmission spread. In some workplaces, respondents reported visible action, mentioning upgraded filters, portable units in classrooms or break rooms, and simple ventilation plans that indicated what kind of filters were used, how often they were changed, and who was responsible for the next check. Workers repeatedly described such transparency as reassuring. A municipal library worker in a unionized setting said that seeing “a handwritten log on the mechanical room door with dates and initials” and hearing “the portable unit humming in the corner” made her feel that “the building was finally on our side.” Another respondent noted that once the joint committee obtained a short explanation from maintenance about how outside air was increased during occupied hours, “arguments about opening windows mostly stopped because we could see someone was watching the air.”

These accounts align with building science guidance that emphasizes clear targets for clean air delivery and regular maintenance and that encourages simple ways to verify performance (Bazant and Bush 2021; Public Health Ontario 2022; Marr and Samet 2024). They also show how joint committees and stewards can translate technical concerns into concrete checks, for example, confirming that units are running and filters are changed on schedule.

In this sample, other respondents described workplaces where ventilation remained invisible, which we term opaque air regimes. Several respondents reported that when they asked about air exchange or filtration, they were told only that “the system meets code” or that “engineering has approved it.” One worker remarked that “hearing we passed inspection last year does not tell me if this room is safe today.” A call center worker described “wipes and posters everywhere and no one who can answer how long the air hangs around.” Workers in these settings often questioned whether the building was safe and sometimes framed infections or exposures as signs of management neglect, a perception that aligns with calls to treat indoor air as part of life safety rather than a background feature of building operations (Morawska and Milton 2020; Public Health Agency of Canada 2024; Hall and Tucker 2022).

The contrast between visible and opaque ventilation highlights the material dimension of safety regimes. Where ventilation routines were documented and visible, workers more often described demanding rules such as masking for long shifts. Where ventilation was unseen and unexplained, surface cleaning and signage were interpreted as thin substitutes for more meaningful controls and became flashpoints for distrust.

### Paid Sick Leave, Precarity, and Presenteeism

Across both union and non-union workplaces, respondents repeatedly described access to paid time off as central in their accounts of decisions about attending work during symptoms or exposures. Workers who reported having paid sick days or other paid leave and who believed they could use them without reprisal described different decision-making than those without such entitlements. A unionized long-term care worker explained that she stayed home with mild respiratory symptoms early in the pandemic because “I knew my sick days would cover me and my manager kept telling us not to risk it.” She added that once the union distributed a leaflet that spelled out that existing sick days could be used while waiting for test results, “people stopped dragging themselves in half sick.”

In this sample, workers without paid sick leave or with very limited entitlements offered a different picture and described what we call income-tested safety. Many reported working while mildly symptomatic, delaying testing or isolating only after a positive result because they could not afford to miss shifts. A non-union restaurant worker wrote that she had “zero paid sick days” and that management discouraged staff from calling in sick unless they had a confirmed test result. Another server recalled being told to use vacation time for isolation and said she had “stopped looking for tests unless I felt really awful” because she could not afford to burn her only week off. A grocery worker described the message as “stay home if you are sick but only if you can pay for it yourself.”

Open-ended comments repeatedly linked the availability and usability of paid leave to decisions about working while ill or exposed. Respondents referenced employer policies as well as federal sickness/income supports that emerged during 2020, and described uncertainty about eligibility, documentation, and whether staying home would create immediate income loss. Unionized respondents more often described union communications or bargaining that clarified how existing sick leave could be used for COVID-related absences and, in some cases, described additional paid days. Non-union respondents more often described ad hoc arrangements that depended on individual supervisors or that required workers to exhaust vacation days before taking unpaid leave. These patterns echo Canadian research on the uneven distribution of sick leave and the policy debates surrounding pandemic-related paid time off (MacIsaac and Morissette 2023; Tucker and Vosko 2021; Government of Ontario 2024).

The interaction between paid leave and employment precarity was particularly stark for workers in temporary, casual, or agency positions. A temporary worker in manufacturing noted that permanent staff had sick days under the collective agreement, but agency staff doing the same work “had none and were not invited when the committee did walk throughs.” She said that she “tried not to rock the boat” because “there were always more temps waiting.” A casual education worker similarly reported that because her hours were not guaranteed she felt unable to refuse in person assignments even when a household member was high risk, explaining that “if I say no too often, I stop getting calls.” An agency worker in logistics described her strategy as “keep your head down and hope you do not get sick” because she felt easy to replace. These accounts echo research on fissured workplaces where layered contracting arrangements limit the reach of union agreements and health and safety protections and concentrate vulnerability among workers with the least leverage (Weil 2014; Vosko, Tucker and Casey 2019).

### Configurations of Safety Regimes and Mental Health

In combination, the survey and comments point to several recurring safety regimes. At one end of the spectrum were more protective regimes in workplaces where infection control routines were visible and predictable, paid sick leave was accessible, and joint committees or worker representatives had time and authority to follow up on concerns. Workers in these settings described high stress due to workload and worry about community transmission but generally felt that management and unions were pulling in the same direction. A hospital worker wrote that she was “tired all the time and scared of another wave” but also that “at least the rules feel real and when we raise something it ends up on a checklist somewhere.” In these workplaces, the material, temporal, and voice dimensions reinforced one another.

At the other end were less protective regimes in workplaces where masking and distancing rules shifted frequently, ventilation was opaque, leave was scarce or difficult to use, and there was little evidence of committee activity or organized worker voice. Workers in these settings reported high anxiety, frequent conflict over rules, and a sense that they were largely on their own. A warehouse worker noted that “every supervisor had a different idea of the rules so the safest plan was to look after yourself and hope for the best.” Another respondent in retail said that “the public message was stay home if you are sick, but the store message was we need you if you are not in the hospital.” In respondents’ accounts, these regimes pushed risk and responsibility down to individual workers.

Many respondents experienced intermediate regimes. Some unionized workers reported strong voice structures but uneven implementation of infection control measures due to chronic staffing shortages. Others had visible ventilation upgrades and clear masking rules but little access to paid leave beyond the provincial program. Some non-union workers reported supportive supervisors who allowed informal flexibility or improvised rotations to reduce exposure and to accommodate isolation, yet these arrangements felt discretionary rather than secure. One worker who held two jobs observed that “in my union job there was a plan on the wall, in my other job there was only a poster that said stay safe.” These hybrids show that safety regimes are not determined by any single factor. They emerge from how material controls, temporal security, and voice intersect in particular workplaces and from how precarity shapes workers' ability to use protections that exist on paper.

In respondents’ accounts, differences between these regimes were salient for mental health and daily experience. Respondents in better-resourced regimes still reported fatigue and worry but more often felt able to focus on core tasks because they trusted that colleagues would stay home when ill, that ventilation and filtration were being maintained, and that disputes about rules could be resolved through known channels. Respondents in thinner regimes described a constant background of vigilance. They checked co-workers' mask use, monitored room occupancy, scanned for open windows, and worried about household members. Several described what one worker called “a second job of risk management” layered on top of paid work. This sense of ongoing hazard featured prominently in reports of anxiety, sleep disruption, and conflict within work teams, and it aligns with wider evidence that perceived adequacy of protections corresponds to mental health symptoms during COVID-19 (Smith et al. 2020; Smith et al. 2022).

Overall, the findings suggest that in this sample, union coverage, ventilation practices, and paid sick leave did not operate as isolated variables. They appeared to combine into more or less protective safety regimes that were reflected in how respondents described their experience of COVID-19 at work and how much of the cost of protection they felt they had to carry themselves.

## Discussion

The findings offer one account of how unions and workplace institutions featured in workers’ accounts of COVID-19 protection in Ontario in this period. In this sample, union coverage mattered less as a simple yes or no and more as one element in a broader configuration of safety regimes. Where union representation was paired with respondents’ descriptions of active joint committee/representative involvement, visible infection control measures, and usable paid leave, workers described clearer rules, more predictable enforcement, and more confidence that concerns would be investigated and resolved. Where any of these elements were missing, union presence alone did not consistently coincide with lower anxiety and conflict in respondents’ accounts.

Although the empirical setting is Ontario, many labor markets rely on comparable internal-responsibility arrangements and employer discretion in translating safety guidance into routine practice. The safety regime lens is intended to travel across these contexts by focusing on institutional configurations rather than a single variable such as union coverage. The Ontario case illustrates, in respondents’ accounts, how the practical meaning of protection was tied to whether material controls were visible and credible, whether workers can afford to comply with public health guidance through paid time off, and whether collective voice can turn guidance into enforceable routines, particularly where precarious employment and contracting chains weaken workers’ leverage. Read this way, the findings generate hypotheses for comparative labor research on worker protection in crises, including when and why collective representation matters most, and how gaps in paid leave and building-level controls shift the costs of protection onto workers and households.

For labor studies more broadly, the Ontario case points to testable propositions: that voice may be most protective when it converts guidance into routines; that paid sick leave may function as compliance infrastructure; that fissuring/casualization may undercut protections even inside union settings; and that ventilation transparency may operate as a trust mechanism by making safety legible and reducing contestation.

Viewed through a worker voice lens, respondents’ accounts are consistent with classic arguments that unions are most effective when they translate individual concerns into enforceable routines rather than isolated complaints (Hirschman 1970; Freeman and Medoff 1984; Walters and Nichols 2007). In the pandemic context, this translation took concrete forms such as checklists, posted protocols, scheduled walk-through inspections, and agreed escalation paths. These devices turned abstract public health guidance into tangible workplace practices and made the internal responsibility system more legible to workers and supervisors alike (Hall and Tucker 2022). When a hospital cleaner could “point to the paper and say here is what has to be done and who did it,” the committee was doing what internal responsibility models assume in principle, namely, converting participation into routine oversight.

At the same time, the analysis underscores the limits of voice in the absence of material supports. Even in unionized settings with active committees, chronic understaffing and limited paid leave strained the system. Workers described choosing between maintaining staffing levels and following recommended isolation periods, and some reported coming to work while mildly symptomatic because they did not want to burden co-workers or be seen as unreliable. These tensions reflect long-standing debates in labor studies about the relationship between production pressures and safety (Weil 2014; Barnetson 2010). The pandemic amplified these dynamics by tying staffing not only to productivity but also to the continued operation of health and education systems. A regime can therefore appear comprehensive on paper yet feel fragile in practice if workload and staffing leave little room to use the protections that exist.

The role of paid sick leave emerges as particularly central in this picture. The data are broadly consistent with wider evidence that paid sick days are not only a matter of individual welfare but also an important part of collective infection control (Pichler, Wen and Ziebarth 2020; MacIsaac and Morissette 2023; Statistics Canada 2024*a*). Workers with dependable paid sick leave described staying home early in the course of illness or exposure, and union communications that clarified how existing sick days could be used were often described as shifting behavior. By contrast, workers without paid leave described income-tested safety, where the practical meaning of public health guidance depended on whether a worker could forgo income. The statement that “the rule was stay home if you are sick but only if you can pay for it yourself” captures this dynamic. For workers in precarious jobs, the absence of paid leave turned guidance into a moral and financial test, not a shared public health practice. Viewed together with this wider evidence, our findings are consistent with arguments that future bargaining and legislation should treat paid sick leave as core public health infrastructure rather than a discretionary benefit.

The attention workers paid to ventilation and indoor air quality speaks to the evolving understanding of airborne transmission and adds a building systems dimension to debates about occupational health. For many respondents, seeing portable air cleaners running, filters being changed on schedule, or simple ventilation plans posted on bulletin boards was described as reducing conflict and anxiety. These practices align with emerging guidance that emphasizes clear targets for clean air delivery and transparent reporting of building performance (Bazant and Bush 2021; Public Health Ontario 2022; Marr and Samet 2024). They also point to a further frontier for union action. Joint committees and stewards can incorporate basic ventilation checks into routine inspections, negotiate for accessible summaries of building performance, and push employers and public authorities to treat indoor air as seriously as other life safety systems such as fire protection (Morawska and Milton 2020; Public Health Agency of Canada 2024). In the terms used here, visible ventilation routines were described as reinforcing safety regimes by strengthening workers’ sense that the building was doing its part.

The safety regime concept helps connect these elements and provides one way to interpret why some workplaces were described as more conflictual and anxiety-producing than others. Two patterns that emerge from the data, which we describe as income-tested safety and opaque air regimes, illustrate how this framework extends existing debates on precarious employment and ventilation by naming the concrete ways that leave policies and building systems shape workers’ sense of protection. In respondents’ accounts, regimes with strong material controls, temporal security, and voice were described as making it easier to align behavior with public health advice without bearing the full cost individually. In these settings, workers still reported fear and exhaustion but less daily contestation over rules. Regimes with weak controls, little paid leave, and limited or suppressed voice were described as shifting risk and responsibility onto workers. In this sample, less protective regimes were not confined to non-union or small workplaces. They also appeared in parts of large organizations where contracting chains, casual positions, or managerial hostility limited the reach of collective agreements and joint committee decisions. This pattern echoes research on fissured workplaces that shows how layers of subcontracting and differential status within the same worksite undermine labor standards even in regulated sectors (Weil 2014; Vosko, Tucker and Casey 2019).

The study has important limitations. The survey sample is a convenience sample and over-represents union members and public sector workers while under-representing racialized workers and those without reliable internet access. Self-selection through labor and community networks may have amplified responses from workers with higher engagement or more salient experiences of workplace conflict or protection. We do not claim that the percentages reported describe all Ontario workers or that the reported differences between groups in this sample are causal; our aim is analytic generalization, identifying mechanisms and configurations plausibly at work in many settings (Smith et al. 2015). The alignment between these within-sample patterns, sector-specific studies, and national indicators on paid sick leave and working from home suggests that the safety-regime framework is a plausible lens for interpreting worker protection during respiratory pandemics and warrants testing with representative and longitudinal data (Buchan et al. 2022; MacIsaac and Morissette 2023; Statistics Canada 2024*a*; Statistics Canada 2024*b*; Sritharan et al. 2025). Nonetheless, future research using representative samples, longitudinal designs, and multilevel methods is needed to test the durability and distribution of these regimes and to examine how they evolve as formal COVID measures recede.

Despite these limits, the study offers several contributions for scholars and practitioners. For labor studies, it underscores the value of integrating occupational health, public health, and employment relations perspectives in the analysis of crisis periods and uses the safety regime lens to move beyond simple union and non-union comparisons. For unions and worker organizations, it highlights concrete strategies that members identified as helpful, including checklists tied to joint committee processes, visible ventilation routines, and proactive communication about how to use sick leave. For employers and regulators, it shows that workers interpret safety not only through formal policies but through everyday routines that signal whether the building and the institution are doing their part and whether protections are usable in practice. The typology of more protective, less protective, and intermediate regimes offers a way to diagnose where institutions are failing and where modest changes in transparency, leave policy, or committee practice could have large effects on how safe work feels.

## Conclusion

The COVID-19 pandemic exposed and intensified long-standing inequalities in work, health, and security. For workers in Ontario, it turned the abstractions of occupational health regulation, paid leave policy, and union representation into immediate questions about whether workplaces could keep people safe and whether workers could protect themselves and their families without sacrificing their livelihoods. Viewing these dynamics through the lens of safety regimes highlights how material controls, temporal security, and collective voice co-occurred and, in respondents’ accounts, contributed to what protection looked and felt like at work.

In this sample, union coverage and usable paid sick leave often co-occurred, and open-ended accounts in these settings frequently referenced active joint committee/representative involvement alongside clearer routines and greater confidence that concerns would be documented and acted on, even amid high workloads and uncertainty. Many others, especially in precarious or contracted positions, described less protective regimes in which infection control was inconsistent, leave was scarce, and voice was constrained. These configurations were salient in how workers interpreted public health guidance, whether they stayed home when ill, and how they managed the daily strain of pandemic work.

Several practical implications follow. Unions can push for paid sick leave and clear language that makes provisions usable without penalty, and can integrate basic ventilation and filtration checks into JHSC routines while ensuring temporary and agency workers are included in safety communications and walk-throughs. Employers can treat indoor air and paid leave as core elements of the duty of care by publishing short rule summaries and maintenance/change logs and investing in transparent ventilation upgrades. Policymakers can establish a modest floor of paid sick days, strengthen enforcement in fissured workplaces, and support small employers with practical templates and resources for indoor air improvements (Pichler, Wen and Ziebarth 2020; Public Health Ontario 2022; Government of Ontario 2024; Public Health Agency of Canada 2024).

For future research, the safety regime framework offers a way to compare workplaces and sectors beyond the specific context of COVID-19. Seasonal respiratory viruses, wildfire smoke, heat waves, and other climate-related hazards will continue to test the capacity of workplaces to protect workers. Studying how material controls, temporal security, and voice interact across these hazards can help identify which institutional combinations are most protective and how unions and other worker organizations can shape them. The experiences of Ontario workers in the first year of the pandemic offer one useful point of reference for this agenda and suggest that modest, well-designed routines may make a meaningful difference in how safe work feels and how the risks of essential services are experienced and distributed.
